# Quantitative Phosphoproteomic Analysis Provides Insight into the Response to Short-Term Drought Stress in *Ammopiptanthus mongolicus* Roots

**DOI:** 10.3390/ijms18102158

**Published:** 2017-10-17

**Authors:** Huigai Sun, Bolin Xia, Xue Wang, Fei Gao, Yijun Zhou

**Affiliations:** College of Life and Environmental Sciences, Minzu University of China, Beijing 100081, China; sunhuigai66@163.com (H.S.); xiabolin8989@126.com (B.X.); wangxue@muc.edu.cn (X.W.)

**Keywords:** phosphoprotein, phosphoproteomics, iTRAQ (Isobaric tags for relative and absolute quantitation), drought stress, *Ammopiptanthus mongolicus*

## Abstract

Drought is one of the major abiotic stresses that negatively affects plant growth and development. *Ammopiptanthus mongolicus* is an ecologically important shrub in the mid-Asia desert region and used as a model for abiotic tolerance research in trees. Protein phosphorylation participates in the regulation of various biological processes, however, phosphorylation events associated with drought stress signaling and response in plants is still limited. Here, we conducted a quantitative phosphoproteomic analysis of the response of *A. mongolicus* roots to short-term drought stress. Data are available via the iProx database with project ID IPX0000971000. In total, 7841 phosphorylation sites were found from the 2019 identified phosphopeptides, corresponding to 1060 phosphoproteins. Drought stress results in significant changes in the abundance of 103 phosphopeptides, corresponding to 90 differentially-phosphorylated phosphoproteins (DPPs). Motif-x analysis identified two motifs, including [pSP] and [RXXpS], from these DPPs. Functional enrichment and protein-protein interaction analysis showed that the DPPs were mainly involved in signal transduction and transcriptional regulation, osmotic adjustment, stress response and defense, RNA splicing and transport, protein synthesis, folding and degradation, and epigenetic regulation. These drought-corresponsive phosphoproteins, and the related signaling and metabolic pathways probably play important roles in drought stress signaling and response in *A. mongolicus* roots. Our results provide new information for understanding the molecular mechanism of the abiotic stress response in plants at the posttranslational level.

## 1. Introduction

As sessile organisms, plants frequently suffer from adverse environmental factors in their lifetimes. Of the various environmental stresses, drought is one of the factors that negatively affects plant growth and development, as well as crop yields and quality. With the ever-increasing world population, rapid development of the economy, and global climate change, the semi-arid and arid regions, worldwide, cover a substantial portion of the land area [1]. Thus, it is of significance to elucidate the plant responses to drought stress and the underlying molecular mechanisms.

To survive and complete lifecycles in the stressful environment, plants have evolved a series of defense and adaptation mechanisms to reduce the damage caused by the water deficiency stress. In recent years, extensive studies using transcriptomic and proteomic approaches have been performed to investigate the plant responses to drought stress, and these works led to the identification of a large number of stress-responsive genes/proteins [2,3]. Further characterization of these stress-responsive genes/proteins promotes our understanding of the abiotic stress regulatory networks in plants. Now, it is commonly accepted that upon perception of the stress signal by some stress sensors, the stress signal may be transmitted via multiple signal cascades, including the mitogen-activated protein kinase (MAPK) pathway. Reactive oxygen species (ROS) and Ca^2+^ are believed to function as two important signaling molecules in stress signaling and plant hormones, in particular, abscisic acid (ABA), also play crucial roles in stress signaling. Stress signaling in plants results in extensive biochemical and physiological alteration, as well as gene regulation.

Protein phosphorylation is one of the most important post-translational protein modifications in cells, and the reversible phosphorylation of proteins regulates many aspects of cell activities [4]. In particular, research showed that protein phosphorylation modification plays an important role in the response to abiotic stress by regulating stomatal switching [5], and defense and stress response in plants [4]. In recent years, large-scale protein phosphorylation analyses have been performed to investigate the protein phosphorylation-medicated abiotic stress response in rice [6], *Arabidopsis thaliana* [7], maize [8], *Brachypodium distachyon* [9], and wheat [10]. However, these phosphoproteomic analyses were performed in model plants and crops, and far less study has focused on non-model plants.

*Ammopiptanthus mongolicus*, an ecologically important shrub in mid-Asia desert regions, exhibits high levels of drought and low-temperature tolerances and is a rare evergreen broad-leafed shrub in the northwest desert of China. *A. mongolicus* has been increasingly used as a model in abiotic tolerance research in trees [11]. The transcriptome analyses of drought stress responses, and miRNA identification and profiling under drought stress, were carried out in *A. mongolicus* [11,12,13,14]. These studies promote our understanding of the molecular mechanisms underlying the drought stress response in *A. mongolicus* and provide important nucleotide and amino acid sequence data for study at the protein level in *A. mongolicus*.

The root is the organ of the plant that takes up water and nutrients from the soil. At the same time, the root probably plays a key role in stress perception, signal transduction, and stress response in plants under drought stress. In the present study, we performed a quantitative proteomic analysis of the response of *A. mongolicus* roots under drought stress, using TiO_2_ enrichment and Liquid chromatography–tandem mass spectrometry (LC–MS/MS) analysis, coupled with Isobaric tags for relative and absolute quantitation (iTRAQ) labeling quantification. A large number of phosphorylation sites and phosphoproteins involved in the drought stress response were identified. These drought-corresponsive phosphoproteins, and the related signaling and metabolic pathways, probably play important roles in drought stress signaling and response in *A. mongolicus* roots.

## 2. Materials and Methods

### 2.1. Material and Stress Treatment

The seeds of *A. mongolicus* were collected in Zhongwei city, Ningxia autonomous district, China. The seeds were surface sterilized using 70% (*v*/*v*) ethanol for 1 min, followed by bleaching (10%) for 6 min, and then rinsed with distilled water thoroughly and germinated on moistened filter paper for 48 h at 25 °C. The seeds were planted in a 30 cm diameter pot containing a 3:1 (*v*/*v*) mixture of vermiculite and perlite. Seedlings were grown in a greenhouse under 120 μmol m^−2^ s^−1^ photosynthetic photon flux density, with a photoperiod of 16 h light and 8 h dark cycle, at approximately 25 °C and 35% relative humidity. The seedlings were watered every four days with half-length Hoagland solution. Eight weeks after germination, the seedlings were subjected to drought treatment.

For drought stress treatments, the seedlings were randomly divided into two groups. The drought treated group was irrigated with 20% PEG-6000 for 1 h, whereas the unstressed group was used as the control. All root samples of the two groups were collected, snap-frozen in nitrogen, and stored at −80 °C until further processing. Four biological replicates were used for iTRAQ labeling in the control or drought stress-treated group, and each biological replicate in the control or drought stress-treated group was pooled from 5 to 8 seedlings.

### 2.2. Protein Extraction

Approximately 0.5 g of fresh root from each biological replicate were ground into a fine power in liquid nitrogen in a mortar. The power was transferred into an acetone solution containing 10% trichloroacetic acid and 65 mM Dithiothreitol (DTT) in a 50 mL tube, and incubated at −20 °C for 1 h. After centrifugation at 17,000× *g* for 45 min, the resulting supernatant was discarded and the pellet was air-dried. The pellet was then suspended in STD buffer (4% SDS, 1 mM DTT, 150 mM Tris-HCl, pH 8.0) and mixed thoroughly by vortexing. After 3 min incubation in boiling water, the suspension was sonicated, incubated in boiling water for another 5 min and then clarified by centrifugation. The protein concentration was determined by the bicinchoninic acid protein assay kit (Beyotime, Beijing, China). The quality of the protein samples were further checked by Sodium dodecyl sulfate polyacrylamide gel electrophoresis (SDS-PAGE) electrophoresis.

### 2.3. Protein Digestion and iTRAQ Labeling

Protein digestion was performed according to the filter-aided sample preparation (FASP) procedure described by Wisniewki et al. [15] and Hu et al. [16]. The peptide content was estimated by spectra density using UV absorption at 280 nm. The resulting peptide mixture was labeled using the 8-plex iTRAQ reagent according to the manufacturer’s instructions (AB SCIEX) (113 to 116 tags for the four biological replicates of the control group, 117 to 121 tags for the four biological replicates of the drought stress-treated group) and vacuum dried.

### 2.4. Enrichment of Phosphorylated Peptiedes by the TiO_2_ Beads

The vacuum-dried peptide mixture was re-suspended in 1× DHB buffer, then added to the appropriate amount of TiO_2_ beads and agitated for 40 min. The sample was centrifuged for 1 min at 5000× *g*, resulting in the first set of beads. The supernatant from the first centrifugation was mixed with more TiO_2_ beads, which were treated before, resulting in the second set of beads. Both sets of beads were pooled and washed three times with wash buffer I (30% ACN/3%TFA, 50 μL) and then three times with washing buffer II (80% ACN/0.3% TFA, 50 μL). Finally, the phosphopeptides were eluted with elution buffer (40% ACN/15% NH_4_OH), lyophilized and re-suspended in 20 μL 0.1% formic acid. Five microliter peptide solution was used for mass spectral analysis.

### 2.5. Mass Spectrometry

Peptide separation was performed with an automated Easy-nLC1000 system coupled to a Q-Exactive mass spectrometer (Thermo Finnigan, San Jose, CA, USA). The enriched phosphopeptides were loaded onto a Thermos Scientific (Rockford, IL, USA) EASY columm (2 cm long, 100 μm inner diameter, RP-C18, 5 μm) equilibrated with 95% Buffer A (Buffer A, 0.1% formic acid), and then the peptides were loaded and separated on a C18 column (75 μm long, 250 mm inner diameter, RP-C18, 3 μm) at a flow rate of 250 nL/min. The peptides were eluted with a gradient of 0–55% buffer B (0.1% formic acid in acetonitrile) from 0 to 220 min, 55–100% buffer B from 220 to 228 min, and 100% buffer B from 228 to 240 min.

For MS data acquisition, the peptides were analyzed in positive ion mode at a resolution of 70,000 (at 200 *m*/*z*). The data-dependent top 10 MS/MS were selected by dynamically choosing the most abundant precursor ions from the survey scan (300–1800 *m*/*z*) using high-energy collision dissociation (HCD). Determination of the target value is based on predictive automatic gain control (pAGC). The dynamic exclusion duration was 25.0 s. Survey scans were acquired at a resolution of 17,500 at *m*/*z* 200. Normalized collision energy was 29 eV and the under-fill ratio, which specifies the minimum percentage of the target value likely to be reached at maximum-fill time, was defined as 0.1%. The instrument was run with peptide recognition mode enabled.

### 2.6. Data Analysis

Raw MS/MS data were interpreted with Proteome Discoverer (version 1.4, Thermo Fisher Scientific, Waltham, MA, USA) using the search engine Mascot (Version 2.2, Matrix Science, London, UK) against the protein database of *A. mongolicus* reported previously [14] and the decoy database. The Mascot parameters were set as follows: enzyme, trypsin; mass values, monoisotopic; peptide mass tolerance, ±20 ppm; MS/MS tolerance, 0.1 Da; max missed cleavages, 2; fixed modifications, carbamidomethyl (C), iTRAQ8plex (N-term), iTRAQ8plex (K); variable modifications, oxidation (M), phospho (ST), and phospho (Y). The decoy database pattern was set as the reverse of the target database. All reported data were based on 99% confidence for peptide identifications as determined by a false discovery rate (FDR) of ≤0.01. Protein identification was supported by at least one unique peptide identification. Phospho RS scores above 50 and PhosphoRS site probabilities above 75% indicate that a site is truly phosphorylated [17].

### 2.7. Bioinformatics

The biological functions of the identified proteins were classified according to their gene ontology annotations and their annotation in KEGG (Kyoto Encyclopedia of Genes and Genomes) database. Protein-protein interaction networks were analyzed using the program STRING (http://string-db.org/), a database of known and predicted protein-protein interactions. The confidence score was set at the medium level (≥0.400).

For conservation analysis of the phosphorylation sites in the identified differentially-phosphorylated phosphoproteins (DPPs), sequences of the DPPs were used as queries to BLAST search the *Arabidopsis* protein database TAIR10 (www.arabidopsis.org) to determine the *Arabidopsis* orthologs, then the phosphorylation information of the corresponding S, T, and Y sites in PhosPhAt database (v4.0) [18] were checked manually to determine the extent of the conservation of phosphorylation sites between ortholog pairs from *A. thaliana* and *A. mongolicus.* The thresholds for BLAST were set at an *E*-value < 1 × 10^−10^, and an identity ≥30%.

Motif-x [19] was adapted for the identification of the phosphorylation motifs present in the phophoproteins. We extracted all items of P(S/T)P (6, 6), a phosphopeptide with a length of 13 with six upstream residues and six downstream residues surrounding the p-site. If the p-site was located in the N-terminus or C-terminus of the protein sequence, the phosphopeptide was complemented to P(S/T)P (6, 6) with the necessary number of “*”s instead of any amino acid. Motif-x parameter settings of “pre-aligned”, central S, T, or Y, width = 13, occurrence = 20, and significance = 0.000001 were adopted.

For the analysis of the structure preferences for the p-sites, NetSurfP ver. 1.1 (www.cbs.dtu.dk) [20] with the default parameters was adopted for the prediction of the secondary structures.

### 2.8. Western Blot and qRT-PCR

Western blot was conducted after 12% SDS-PAGE by probing with primary antibodies at the recommended dilutions: anti-HSP70 (1:1000, rabbit polyclonal; Beijing Protein Innovation, Beijing, China), anti-MPK6, and anti-PIP1 (1:2000, rabbit polyclonal; Beijing Protein Innovation, Beijing, China). Fifty to eighty micrograms of proteins were used depending on the sensitivity of the specific antibody. The protein concentration was measured using Bradford methods (Bioteke, Beijing, China). Goat anti-rabbit IgG (H+L) 800 CW (1:5000, LI-COR) was used as the secondary antibody. Visualization and quantification were carried out with the LI-COR Odyssey scanner and software (LI-COR Biosciences).

Quantitative real-time RT-PCR was performed according to a previously described protocol [14]. All primers used in this study are listed in Table S1 AmeIF1 (GenBank accession no. JN885965) was used as the internal reference gene.

### 2.9. Statistical Analysis

The phosphoprotein assays were performed in four biological replicates. Statistical analyses of phosphopeptides were conducted using the Student’s *t*-test with Microsoft Excel (version 2010, Microsoft Corp., Redmond, WA, USA) and a *p*-value ≤0.05 was considered statistically significant.

## 3. Results

### 3.1. Characteristics of Identified Phosphorylation Events in A. mongolicus Roots

To analyze the phosphoprotein composition of *A. mongolicus* roots and its change under short-term water deficient status, we conducted an iTRAQ-based quantitative proteome analysis using the drought treated and the untreated roots. The protein concentration of root protein samples were measured by the Bicinchoninic acid (BCA) method (Table S2) and the quality of protein samples were evaluated by SDS-PAGE analysis.

**Figure 1:**
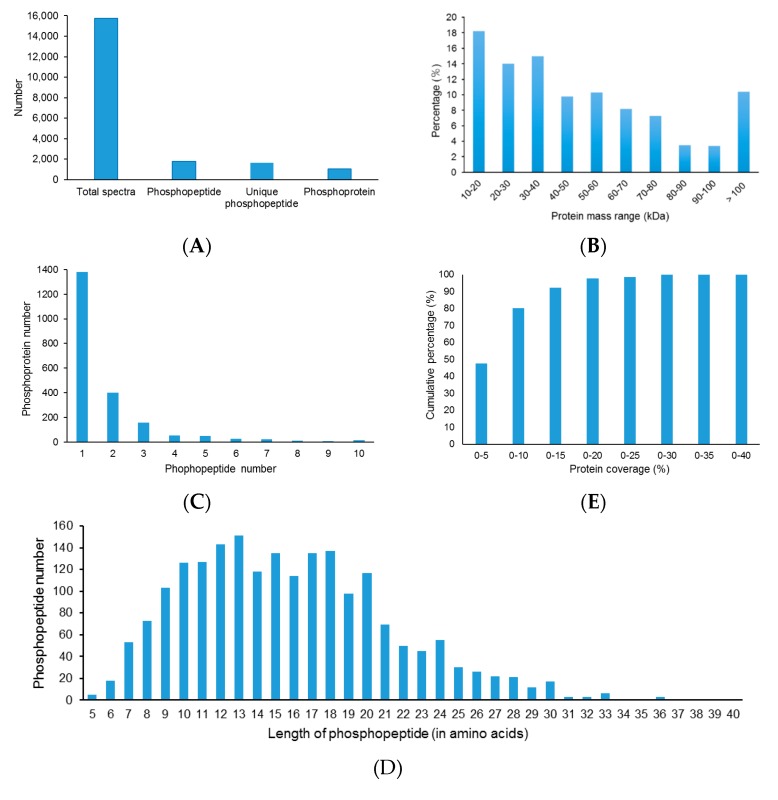

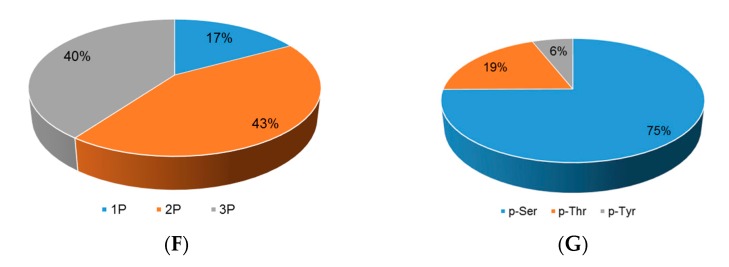
Characteristics of the identified unique phosphopeptides in *A. mongolicus* root samples. (**A**) Basic information statistics; (**B**) protein mass distribution; (**C**) unique peptide number distribution; (**D**) distribution of phosphopeptides based on their length; (**E**) protein coverage; (**F**) distribution of phosphopeptides depending on their number of phosphorylation sites (1P, one phosphorylation sites; 2P, two phosphorylation sites; 3P, three phosphorylation sites or more); and (**G**) the distribution of serine phosphorylation (p-Ser), threonine phosphorylation (p-Thr), and tyrosine phosphorylation (p-Tyr) sites in *A. mongolicus* roots.

A total of 2019 peptides (FDR ≤ 0.01) representing 1646 unique peptides were obtained and 1060 proteins were ultimately identified (Figure 1A, Tables S3 and S4). The predicted molecular weights of the identified proteins vary greatly with molecular weights ranging from 10.9 to 251.7 kDa with a mean of 54.6 kDa (Figure 1B). The distribution of the number (Figure 1C) and length (Figure 1D) of phosphopeptides, and the sequence coverage of phosphoproteins (Figure 1E) were also provided. More than 34.8% of the identified phophoproteins were identified from at least two peptides (Figure 1C). Protein sequences coverage with 30–40%, 20–30%, 10–20%, and under 10% variation accounted for 0.24%, 2.17%, 17.5%, and 80.03%, respectively, of the total identified phosphoproteins (Figure 1E).

In total, 7841 phosphorylation sites were found from the 2019 identified phosphopeptides. Among of these phosphopeptides, 346 peptides contained one phosphorylation site, 869 peptides contained two, and 804 peptides contained three or more phosphorylation sites (Figure 1F). Of the identified phosphorylation sites, most of them (75.27% (5902/7841)) occurred on serine (S) residues, some of them (19.26% (1510/7841)) occurred on threonine (T) residues, and only a few of them (5.47% (429/7841)) occurred on tyrosine (Y) residues (Figure 1G).

### 3.2. Identification of Differentially-Expressed Phosphopeptides and Phosphoproteins under Drought Stress in A. mongolicus Roots

First, we determined the cutoff for up- or down-regulation based on the label-specific experimental variation between the four replicates for the two experimental groups (control: 113–116; stressed group: 117–121). We analyzed the distribution of the fold change (FC) ratio (i.e., stressed group vs. control) (Figure S1). Then the frequency distribution of the fold deviation from the mean of each groups was calculated (Figure S2). The experimental variation was ≤1.3-fold for around 97.41% and 94.66% of the ratios for the control and drought-stressed groups. Based on these results, the cut-off used for identification of the differentially-expressed phosphopeptides (DEPP) was set at 1.3-fold; a ratio >1.30 and <0.77 was considered as up- or down-regulated.

**Figure 2:**
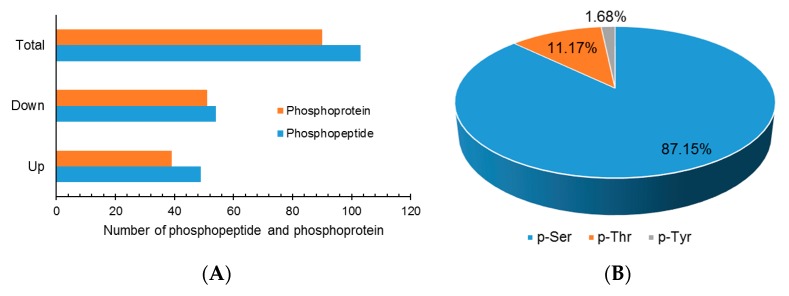
Differentially-expressed phosphopeptides and phosphoproteins in *A. mongolicus* roots under drought Stress. (**A**) Summary of the differentially-expressed phosphopeptides and phosphoproteins; and (**B**) distribution of serine phosphorylation (p-Ser), threonine phosphorylation (p-Thr), and tyrosine phosphorylation (p-Tyr) sites of the differentially-expressed phosphopeptides in *A. mongolicus* roots.

In total, the abundance of 103 phosphopeptides exhibited significant change under drought stress (ratio >1.3 or <0.77, and *p* < 0.05) (Figure 2A). Among these drought stress-induced differentially-expressed phosphopeptides, 49 were up-regulated and 54 were down-regulated under drought stress. In total, 179 phosphorylation sites were identified from the 103 phosphopeptides, and of these phosphorylation sites, 156 (87.15%) were serine (S) residues, 20 (11.17%) were threonine (T) residues, and three (1.68%) were tyrosine (Y) residues (Figure 2B).

The 103 phosphopeptides were cleaved from 90 phosphoproteins, thus these phosphoproteins were deemed as differentially-phosphorylated phosphoproteins (DPPs). Among these DPPs, 82 harbored only one identified DEPP, five harbored two DEPPs, and three harbored three or four DEPPs (Table S5). Based on the change pattern of the phosphopeptides, the phosphorylation levels of 39 DPPs were thought to be increased, and that of 51 DPPs were thought to be decreased.

We further conducted conservation analysis of the phosphorylation sites in the identified DPPs by check if the corresponding amino acids in sequences of the *Arabidopsis* orthologs have been reported to be phosphorylated. The results showed that, of the 179 phosphorylation sites in the identified DPPs, 74 corresponding S, T, or Y sites were present in *Arabidopsis* orthologs, and of these 74 S, T, or Y sites, 51 (68.92%) have been reported to be phosphorylated (Table S6). Thus, these 51 phosphorylation sites were conservative between *Arabidopsis thaliana* and *A. mongolicus*, and the other 23 sites might be novel phosphorylation sites found in *A. mongolicus* under drought stress.

We examined the drought stress-induced alteration in protein abundance for two DPPs (MPK6 and PIP1) using Western blot, however, no significant changes were observed (Figure S3). The gene expression levels of 10 DPPs under drought stress were also investigated by the quantitative reverse transcription PCR (qRT-PCR) method (Figure S4), and the results showed that five of these genes, including MPK6 and PIP1 encoding genes, were up-regulated, and one gene was down-regulated in *A. mongolicus* under drought stress.

### 3.3. Sequence and Structure Features of Phosphorylation Sites in Drought-Induced Differentially-Expressed Phophopeptides in A. mongolicus Roots

**Figure 3:**
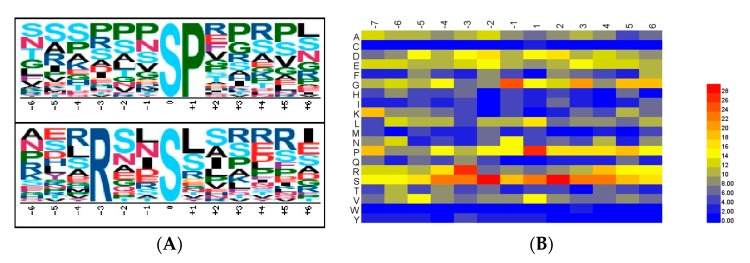

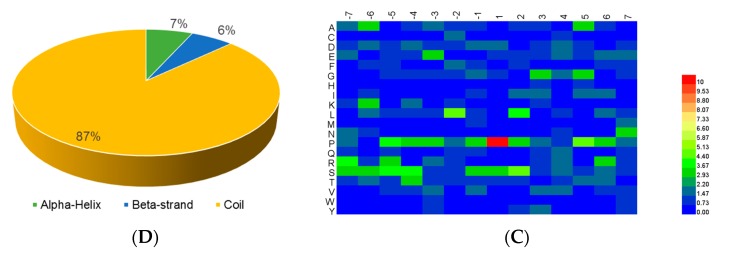
Analysis of phosphorylation sites by sequence motif, the distribution of amino acids in the flanking sequences and structural preferences in the differentially-expressed phophopeptides in *A. mongolicus* roots under drought stress. (**A**) The sequence motif analysis of phosphorylation sites using Motif-x; (**B**) the heatmap for the distribution of amino acids in serine phosphorylation site flanking sequences; (**C**) the heatmap for the distribution of amino acids in threonine phosphorylation site flanking sequences; and (**D**) the secondary structural distribution for the phosphorylation sites.

Each phosphorylation modification associated with specific protein kinase. To identify primary initial protein phosphorylation inducers, we submitted the flanking sequences of high-confident phosphorylation residues identified in the DEPPs from *A. mongolicus* roots to Motif-x [19], an online software tool (http://motif-x.med.harvard.edu/motif-x.html), to extract overrepresented patterns by comparison to a dynamic background. Consequently, of all the DEPPs, two enriched serine phosphorylation-based motifs were identified, i.e., [pSP] and [RXXpS] (Figure 3A). These motifs have been reported to be associated with certain kinases: p[S/T]P for GSK-3, cyclin-dependent kinase (CDK) and mitogen-activated protein kinases (MAPK) families; and RXXpS for the SNF1-related kinase II (SnRK2) family [21,22]. The results indicate that these kinases might play crucial roles in the response to drought stress in *A. mongolicus* roots.

To evaluate the relative frequency of the specific amino acids flanking the phosphorylation sites, the heatmap for the items of P(S/T)P (7, 7), a phosphopeptide with a length of 15, with seven upstream and seven downstream residues around the phosphorylation site, was visualized using HemI [23] (Figure 3B for serine phosphorylation sites, Figure 3C for threonine phosphorylation sites). The P at the +1 position was the amino acid had the highest frequency, and the S at the +2 position was also significantly enriched. It is noteworthy that the high frequency of S was observed in the flanking sequence of both serine and threonine phosphorylation sites and the high frequency of R (arginine) appeared only in serine phosphorylation sites. In general, the results revealed by the heatmap are in line with the motif analyses above.

In addition, we also analyzed the secondary structural preferences for the phosphorylation sites using NetSurfP ver. 1.1 software (www.cbs.dtu.dk) [20], the results indicated that protein phosphorylation predominantly occurred in amino acids with a secondary structure of a coil, which had a coverage of more than 87% in the present study (Figure 3D).

### 3.4. Functional Analysis of the Differentially-Phosphorylated Phosphoproteins in Drought Stressed A. mongolicus Roots

To understand the biological roles of differential protein phosphorylation in response to drought stress in *A. mongolicus* root, we annotated the DPPs by the enrichment analysis in the Gene Ontology (GO) function term and the Kyoto Encyclopedia of Genes and Genomes (KEGG) pathway (Table S7).

**Figure 4:**
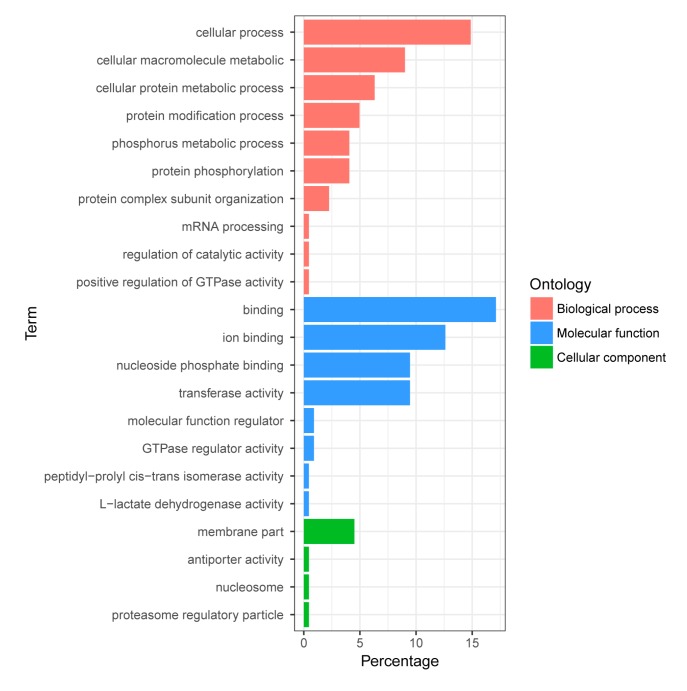
GO enrichment analysis of the differentially-phosphorylated phosphoproteins in *A. mongolicus* roots.

Firstly, we conducted gene GO enrichment analysis for all DPPs. The enriched GO terms associated with 22 GO categories. As shown in Figure 4, the DPPs were classified into 10 groups based on their biological processes, these GO terms included protein phosphorylation, mRNA processing, regulation of catalytic activity, and positive regulation of GTPase activity. The DPPs were clustered into four groups according to their cell component, and these GO terms included membrane part, nucleosome, proteasome regulatory particle, and anti-porter activity. The DPPs were classified into eight groups based on their molecular function, and these GO terms included ion binding, nucleoside phosphate binding, peptidyl-prolyl cis-trans isomerase activity, GTPase regulator activity, and l-lactate dehydrogenase activity.

**Figure 5:**
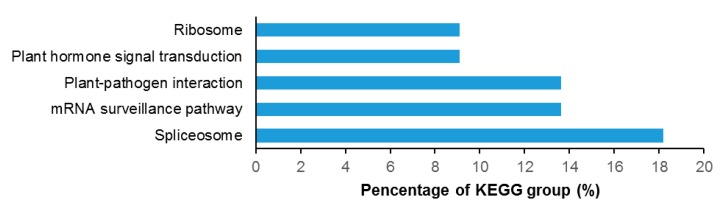
The top five KEGG pathways in the differentially-phosphorylated phosphoproteins in *A. mongolicus* roots under drought stress.

To reveal the metabolism pathways that were involved in drought stress response, the 90 DPPs were further analyzed by using the KEGG database (Figure 5). In total, we identified 13 enriched KEGG pathways in response to drought stress. As shown in Figure 5, many fundamental biological pathways were overrepresented by phosphoproteins identified in this study, including spliceosome, mRNA surveillance pathway, plant-pathogen interaction, plant hormone signal transduction, and ribosome.

All DPPs were further annotated by aligning to the *Arabidopsis* protein database (TAIR10) and Swissprot database. According to the annotation results, together with the GO and KEGG analyses, all DPPs were classified into ten categories, i.e., osmotic adjustment, RNA splicing and transport, protein synthesis, folding, and degradation, stress response and defense, signal transduction and transcriptional regulation, vesicle transport, cell skeleton, epigenetic regulation, carbohydrate metabolism, and miscellaneous and unknown proteins (Table S8). Their possible functions in drought stress signaling and response will be discussed later.

### 3.5. Protein-Protein Interaction Analysis

**Figure 6:**
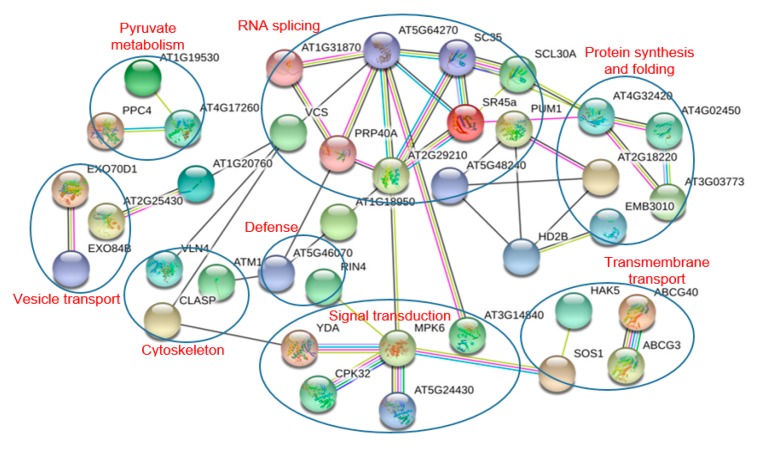
The protein-protein interaction (PPI) network of the differentially phosphorylated phosphoproteins in *A. mongolicus* roots under drought stress. The functional classification of the DPP group are shown in red. Edge color represents protein-protein associations among different proteins: blue for known interactions from curated database, pink for experimentally determined known interactions, green for predicted interactions base on gene neighborhood, red for predicted interactions base on gene fusions, dark blue for predicted interactions base on gene co-occurrence, yellow-green for predicted interactions base on textmining, black for predicted interactions base on co-expression, and light blue for predicted interactions base on protein homology.

In order to reveal the interaction network associated with the drought stress responsive phosphoproteins, a protein-protein interaction (PPI) network was constructed using the STRING protein-protein interaction database, KEGG pathway, and GO biological process analyses (Figure 6). The names of the DPPs were represented by the names or the locus numbers of the homologous proteins in *Arabidopsis* (http://www.arabidopsis.org) in the PPI map.

The largest subnet in the PPI network is composed of 10 RNA splicing-related DPPs. Five signal transduction-related DPPs form another subnet. These DPPs include YDA, MPK6, and a CPK32, and these phosphoproteins might play important roles in drought stress signaling. The other large subnet is composed of five DPPs involved in protein synthesis and folding, including two HSP20s, a PPIase, and a ribosomal protein S6. The other subnets in this PPI network were associated with vesicle transport, transmembrane transport, cytoskeleton, defense, and pyruvate metabolism.

## 4. Discussion

In the present study, we conducted an iTRAQ-based quantitative phosphoproteomic analysis of the response to short-term drought stress in *A. mongolicus* roots. A large number of phosphorylation sites were identified and a batch of phosphoproteins possibly involved in the drought stress response were found. These drought-responsive phosphoproteins and the related signaling and metabolic pathways may play important roles in drought stress signaling and response in *A. mongolicus* roots.

### 4.1. Phosphoproteins Involved in Signal Transduction and Transcriptional Regulation

Protein phosphorylation play a crucial role in signal transduction in plant cell. In the present study, eight kinases were found to be differentially phosphorylated under drought stress and these kinases probably participated in the drought stress signal transduction process in *A. mongolicus* roots.

ABA, a plant hormone, is a key signaling molecule regulating plant responses to abiotic stress. As expected, the motif analysis revealed that many phosphorylation sites in DPPs were binding sites of SnRK2, the key kinases of ABA signaling pathways, highlighting the role of the ABA signaling in drought stress response in *A. mongolicus* roots.

The MAPK cascade is one of the most important signaling pathways working in transmitting cellular signals. The MAPK cascade consists of three tier components: mitogen-activated protein kinase kinase kinases (MAPKKKs), mitogen-activated protein kinase kinases (MAPKKs), and mitogen-activated protein kinases (MAPKs), and the pathway carries out the phosphorylation reaction from the upstream receptor to the downstream target [24]. Two kinases related to MAPK cascades, i.e., YDA and MAPK6, were differentially phosphorylated under drought stress in the present study, indicating that the MAPK pathway is involved in drought stress signaling in *A. mongolicus* roots. YDA has been demonstrated to act upstream of the MKK4/MKK5-MPK3/MPK6 module to regulate stomatal development [25] and, in a recent study, YDA was shown to be regulated at the transcriptional level by AN3 to improve root systems under drought stress [26].

Ca^2+^ participates in signal transduction in plants under environmental stress and calcium-dependent protein kinase (CDPKs) plays a crucial role in the Ca^2+^-mediated signaling pathway. In the present study, The phosphorylation levels of CPK32 (*comp30972_c0_seq1*) and a CDPK (*comp9850_c0_seq1*) were down-regulated under drought stress, indicating the Ca^2+^-mediated pathway participates in drought stress signaling in *A. mongolicus* roots. CPK32 regulates the ABA-responsive gene expression via phosphorylation of the ABA-responsive element (ABRE)-binding transcription factors 4 (ABF4) in *Arabidopsis* [27].

Brassinosteroids (BRs), a group of plant steroid hormones, play important roles in plant growth, development, and stress responses. BR signaling through transmembrane receptor kinases and intracellular cascades leads to dephosphorylation and accumulation of the BZR1 [28]. We found a BZR1 homolog (*comp7746_c0_seq1*), the key transcription factor in the brassinosteroid (BR) signaling pathway were differentially phosphorylated under drought stress in *A. mongolicus* roots. The down-regulated phosphopeptide, ISNs(ph)APVt(ph)PPLs(ph)SPTWR, harboring a typical motif of GSK-3 kinase, the key kinase mediating the BR response by phosphorylate BZR1. This observation suggested that the activity or abundance of GSK-3 may be decreased under drought stress.

In addition to BZR1, some other transcription factors (TFs) were found to be differentially phosphorylated under drought stress, for example, floral homeotic protein (HUA1) was involved in flower development under stressful environments [29]. These TFs may contribute greatly to the gene regulation network in responding to the short-term drought stress.

### 4.2. Phosphoproteins Involved in Transmembrane Transport, Cytoskeleton, and Vesicle Transport

Maintenance of the cell’s osmotic potential under water-deficient conditions is a major challenge for plant growth and development. In the present study, several aquaporins and ion transporters were found to be differentially phosphorylated under drought stress, suggesting protein phosphorylation of these proteins may play roles in regulating osmotic balance upon short-term drought stress in *A. mongolicus* roots.

Aquaporins (AQPs), a family of highly-conserved transmembrane proteins, are involved in the quick transport of water and other small non-polar molecules, and regulation of the osmotic balance. We found a serine phosphorylation site in an up-regulated phosphopeptide: EQDVS(ph)LGANK in a PIP1-1 homolog (*comp29561_c0_seq1*) under drought stress. PIPs were the most abundant aquaporins in the plasma membrane, and most PIPs are expressed dominantly in the root and down-regulated by water stress in *Arabidopsis* [30]. In addition to the transcriptional regulation, PIP1 and PIP2 subgroups were also regulated by phosphorylation and proper phosphorylation can modulate the channel activity of PIPs [31]. We speculated that the differential phosphorylation upon drought stress might be a quick way to regulate activities of aquaporins in *A. mongolicus* roots.

Several molecular pumps were also found to be differentially phosphorylated under drought stress in *A. mongolicus* roots and some of them may contribute to the osmotic adjustment under drought stress. Among these proteins, SOS1 is one of the well-documented phosphoproteins, which is involved in ion transmembrane transport regulated by the SOS signaling pathway [32]. Some of these pumps transport ions, such as sodium (SOS1), calcium (sodium/calcium exchanger family protein), and potassium (high-affinity K^+^ transporter 5); the others transport amino acids and other small molecules (transmembrane amino acid transporter family protein, pleiotropic drug resistance 12, ABC-2 type transporter family protein, and tonoplast monosaccharide transporter 2). The alteration in phosphorylation status of these transporters upon drought treatment indicate their possible roles in regulating ions and small molecule concentrations to reduce water loss from cytoplasm. Such a regulation may not only contribute to osmotic adjustment and nutrient absorption, but also be associated with regulation of Ca^2+^-mediated signaling.

Under the high osmotic environment, water efflux was enhanced and the cell volume became smaller in plant roots [33]. To cope with such stress, the cytoskeleton, cell wall, and plasma membrane would change correspondingly. We found five cytoskeleton-related proteins differentially phosphorylated under drought stress. Of these phosphoproteins, four were related to microfilaments, including villin 2, villin 4, and formin homology 2, and the other phosphoprotein was related to microtubules. Phosphorylation modification of villins and formins were proposed to play roles in modulating their activities in human and animal cells [34,35]. In the present study, the cytoskeleton-related DPPs probably participate in modulating the cytoskeleton organization in drought-stressed root cells. The cell wall is critical for cell shape maintenance and two proteins involved in cell wall formation were regulated at the phosphorylation level. Of these proteins, the cellulose synthase family protein participate in the secondary cell wall formation, and glucan synthase-like 5 plays a role in callose deposition in the cell wall [36].

In addition, five proteins involved in exocytosis and endocytosis were found to be differentially phosphorylated under drought stress. Previous study showed that short-term extracellular osmotic treatments result in a shift in the balance between endocytosis and exocytosis in root meristem cells by influencing the tension on the plasma membrane [37], and we speculated that these five DPPs might contribute to the drought stress-induced alteration in tension on the plasma membrane in *A. mongolicus* roots.

### 4.3. Phosphoproteins Involved in RNA Splicing and Transport

To create translatable mRNAs, pre-mRNA molecules need to be properly processed, spliced, and transported to the cytoplasm. Recent studies showed that post-transcriptional regulation, especially alternative splicing (AS) and mRNA export, is essential for plants to adapt to environmental stress. In the present study, a large number of phosphoproteins involved in the post-transcriptional regulation of RNA were identified, highlighting their important roles in drought stress response in *A. mongolicus* roots.

The SR (serine arginine-rich) proteins are a class of proteins participating in spliceosome assembly in plants. SR proteins consist of one or two RNA recognition motifs (RRMs) that contact the RNA targets and a domain enriched in serine and arginine residues involved in protein-protein interaction. We found three SR proteins whose phosphorylation levels were up-regulated under drought stress, and these SR proteins include SCL30A, SC35, and SR45a. In addition, there are four additional DPPs involved in RNA splicing and the phosphorylation levels of most of these phosphoproteins were increased under drought stress. Phosphorylation is proposed to modulate protein–protein interactions within the spliceosome, thereby contributing to dynamic structural reorganization of the spliceosome during splicing [38]. Given that accumulating evidence supports the idea that AS is a new means of regulating the environmental fitness of plants [39], we speculate that phosphorylation of RNA splicing-related proteins possibly serve as an immediate means to regulate the environmental fitness of plants by modulating the AS process in *A. mongolicus* roots under short-term drought stress.

### 4.4. Phosphoproteins Involved in Protein Synthesis, Folding, and Degradation

Protein synthesis and folding may be disturbed and, hence, protein degradation may be activated in plants under stress condition, and previous proteomic studies have identified a large number of drought-corresponsive proteins associated with protein synthesis, folding, and degradation [40]. In the present study, two DPPs involved in ribosomes and translation, four DPPs related to protein folding, and four DPPs associated with protein degradation were identified, suggesting the important role of protein phosphorylation in regulating protein turnover at the post-transcriptional level.

Ribosomal protein S6 (RPS6), a component of the 40 S ribosomal subunit, has been shown to be a key component of the TOR-S6k- RPS6 signaling pathway [41]. Environmental cues, such as osmotic stress, affect phosphorylation status of RPS6 via TOR pathway and the phosphorylation level of RPS6 is closely associated with cell proliferation activity and cell size [42]. The addition of fresh medium to *Arabidopsis* suspension cell culture promotes phosphorylation of RPS6 by activating the cognate kinase of RPS6, AtS6k. In the present study, six serine phosphorylation sites were identified in an RPS6 homolog (*comp8048_c0_seq1*) and the phosphorylation levels of these phosphorylation sites were down-regulated under drought stress. We speculate that the drought stress signal transmitted by the TOR pathway resulted in down-regulation of RPS6 phosphorylation observed in the present study and the alteration in RPS6 phosphorylation status may influence the root under drought stress.

Heat shock proteins (HSPs) are a family of proteins that are generated by cells upon exposure to stressful conditions and many members of HSPs perform chaperone functions. In the present study, two HSP20-like proteins (*comp41212_c0_seq1* and *comp17846_c0_seq1*) were differentially phosphorylated under drought stress. The stress-induced change in phosphorylation status in small molecular HSPs was also observed in maize [16].

In addition, four proteins involved in protein ubiquitination, and misfolded proteins exporting from the endoplasmic reticulum were found to be differentially phosphorylated under drought stress, indicating protein degradation via the ubiquitination pathway may be regulated by protein phosphorylation upon short-term drought stress in *A. mongolicus* roots. In brief, our study provided data to advance our understanding of the important role of protein phosphorylation in regulating protein turnover at the post-transcriptional level.

### 4.5. Phosphoproteins Involved in Epigenetic Regulation

Epigenetic regulations such as histone modification have been proposed to play key roles in gene regulation in plants under stress [43]. Not surprisingly, three proteins involved in epigenetic regulations were differentially phosphorylated under drought stress in *A. mongolicus* roots. Of these proteins, histone deacetylation mediate transcriptional repression and histone deacetylase 2B (HD2B) may play an important roles in transcriptional regulation in plant [44].

### 4.6. Phosphoproteins Involved in Pyruvate Metabolism

Two enzymes involved in pyruvate metabolism were differentially phosphorylated under drought stress in *A. mongolicus* roots. Phosphoenolpyruvate carboxylase 4 (PPC4, comp36964_c0_seq1) may provide more oxaloacetate used for the synthesis of amino acids, like aspartic acid and glutamic acid, which were observed to accumulate in plant cells under drought stress [45]. PPC4 and malate dehydrogenase (*comp20184_c0_seq1*) can work together to synthesize malate from glycolytically-derived phosphoenolpyruvate.

### 4.7. Gene Expression and Protein Abundance of Selected DPPs under Drought Stress

The alteration in phosphorylation level of an individual protein might have resulted from either the up-regulation of the activity or abundance of the corresponding kinase, or the increase of the protein abundance. The Western blots showed that although the phosphorylation level of MPK6 and PIP1 were up-regulated under drought stress, there were no significant alterations in protein abundance of the two proteins (Figure S3). Thus, the drought stress-induced changes in phosphorylation status of MPK6 and PIP1 were probably caused by the alterations of the activity or abundance of the corresponding kinase.

Not surprisingly, the qRT-PCR analysis showed that drought stressed-induced changes in gene expression levels were not correlated with that of the phosphorylation status. However, it is noteworthy that the MPK6 and PIP1 encoding genes (*comp15811_c0_seq1* and *comp29561_c0_seq1*) were observed to be up-regulated in transcription level (Figure S4), which was different with that of protein abundance level. Many previous studies have reported the inconsistency between gene expression level and protein abundance level [46], and our results indicated that DPPs, like MPK6 and PIP1, were regulated at both the transcription level and post-translational modification level during the first 1 h of drought stress in *A. mongolicus* roots. Taken together, the difference between transcription level, protein accumulation level, and posttranslational level highlighted the complexity of the regulation network involved in plant response to environmental stresses.

## 5. Conclusions

Although extensive studies have been performed in investigating the molecular mechanism underlying the response to drought stress in plants, little is known about how reversible protein phosphorylation functions in the drought stress response. In the present study, we used an iTRAQ-based quantitative phosphoproteomic approach to analyze the short-term drought stress response in *A. mongolicus*, an ecologically important shrub in mid-Aisa desert regions. Of the large amount of phosphopeptides and phosphoproteins identified, 103 DEPPs and 90 DPPs were determined. The motif analysis of the phosphorylation sites in the DEPPs, together with functional and PPI analysis of the DPPs, revealed that signaling pathways, such as MAPK and CPK cascades, and ABA and BR hormone signaling pathways, were involved in stress signal transduction in *A. mongolicus* roots. Our results demonstrated that, as a rapid and reversible means to modulate protein activity, reversible protein phosphorylation mediated adjustment of diverse biological processes were induced by drought treatment, and these biological processes included osmotic adjustment, RNA splicing and export, protein synthesis, folding and degradation, as well as stress response and defense. Thus, this study provides important data for understanding the signal transduction and gene regulation mediated by reversible protein phosphorylation in plants in stressful conditions.

## Supplementary Materials

Supplementary materials can be found at www.mdpi.com/1422-0067/18/10/2158/s1.
